# The Use of Ground Coal Bottom Ash/Slag as a Cement Replacement for Sustainable Concrete Infrastructure

**DOI:** 10.3390/ma17102316

**Published:** 2024-05-14

**Authors:** Sandip Poudel, Samrawit Menda, Joe Useldinger-Hoefs, Lidya E. Guteta, Bruce Dockter, Daba S. Gedafa

**Affiliations:** 1 Red River Valley Alliance LLC, 4816 Amber Valley Pkwy S, Fargo, ND 58102, USA; spoudel@rrvafm.com; 2 Bingham Engineering Consultants, 13416 N. 32nd St. Suite 100, Phoenix, AZ 85032, USA; s.menda@binghameng.com; 3 Michael Baker International, 120 South 6th St. Unit 1710, Minneapolis, MN 55402, USA; joe.useldinger@mbakerintl.com; 4 Department of Civil Engineering, University of North Dakota, 243 Centennial Drive Stop 8115, Grand Forks, ND 58202, USA; lidya.guteta@und.edu (L.E.G.); bruce.dockter@und.edu (B.D.)

**Keywords:** coal bottom ash (CBA), coal boiler slag (CBS), pozzolanic properties, compressive strength, durability, workability, loss on ignition (LOI), Blaine fineness

## Abstract

Cement production requires considerable energy and natural resources, severely impacting the environment due to harmful gas emissions. Coal bottom ash (CBA) and coal boiler slag (CBS), byproducts of coal-fired powerplants having pozzolanic properties, can be mechanically ground and replace cement in concrete, which reduces waste in landfills, preserves natural resources, and reduces health hazards. This study was performed to determine the optimum cement replacement amount of ground CBA (GCBA) and ground CBS (GCBS) in concrete, which was 10% for GCBA and 5% for GCBS. GCBA-based concrete exhibited superior tensile strength, modulus of elasticity, and durability compared to the control. In the Rapid Chloride Penetration Test, 10% GCBA concrete resulted in 2026 coulombs at 56 days, compared to 3405 coulombs for the control, indicating more resistance to chloride penetration. Incorporating 2.5% nanoclay in GCBA-based concrete increased the optimum GCBA content by 5%, and the compressive strength of 15% GCBA concrete increased by 4 MPa. The mortar consisting of the finest GCBA(L1) having Blaine fineness of 3072 g/cm^2^ yielded the highest compressive strength (32.7 MPa). The study discovered that the compressive strength of GCBA and GCBS-based mortars increases with fineness, and meeting the recommended fineness limit in ASTM C618 enhances concrete or mortar properties.

## 1. Introduction

Concrete is one of the most widely used building materials due to its simplicity and availability, and cement is an expensive material used in production [1]. Approximately 4.3 gigatons of cement were produced in 2020, according to the International Energy Agency (IEA). Carbon dioxide emissions from cement production increased by 1.8% annually from 2015 to 2020 [2]. Cement production requires a significant amount of natural resources and energy, which significantly impacts the environment due to harmful gas emissions. Approximately 600 kg of cement production releases 400 kg of carbon dioxide [3]; finding a binding material to replace cement is critical for long-term sustainability.

Nowadays, researchers are exploring various cementitious and recycled materials to enhance concrete properties, aiming for improved sustainability and performance [4,5,6]. Research and development on 3D printing concrete using fly ash and blast furnace slag as mineral additives could address economic and environmental challenges in the future due to the construction industry [7]. Golewski [8] studied the effect of siliceous fly ash, silica fume, and nanosilica on cement matrix morphology and microcrack size in concrete. The paper reveals that adding pozzolanic additives leads to a more uniform structure and reduces microcrack width, enhancing concrete durability. Xiao et al. [9] explored the impact of incorporating siliceous fly ash, silica fume, and nanosilica on cement matrix morphology and microcrack size in concrete, revealing improved homogeneity and reduced microcrack width with pozzolanic additives.

Coal bottom ash (CBA) and coal boiler slag (CBS) are byproducts of coal-fired power plants. The properties of these byproducts depend on the furnace type used by the power plant [10]. The oven-dried CBA or CBS are mechanically ground to obtain ground coal bottom ash (GCBA) or ground coal boiler slag (GCBS); the physical properties vary by fineness. GCBA has good pozzolanic properties, which increase as the fineness increases [11,12,13]. The researchers have revealed that locally sourced untreated CBA demonstrates promising potential in mitigating ASR expansion within concrete structures [14]. Also, unburnt CBA bricks have a potential alternative to conventional burnt clay bricks [15].

Ganesan et al. [16] studied the use of coal bottom ash (CBA) as a cost-effective alternative to partially replace fine aggregate, combined with ultra-fine slag material (Alccofine), as a partial cement replacement. Although a notable decrease in strength properties was noticed with increasing CBA concentrations from 10% to 50%, the Alccofine addition significantly improved workability and strength by reducing void space in concrete. Concrete mix with 40% CBA and 15% Alccofine demonstrated peak strength properties. Rapid Chloride Penetration Test (RCPT) revealed low chloride-ion penetration in the concrete mix due to its dense pore structure, affirming its desirability. SEM analysis confirmed the formation of extra C-S-H and dense pore structures contributing to higher strength gain. Even though the cost of concrete increased by around 8% with the addition of Alccofine, the compressive strength increased by 58%, which is significant.

Chuang et al. [17] investigated the potential of GCBA to replace Portland cement in concrete with coal fly ash from the same power plant. Incorporating GCBA required the addition of a superplasticizer to achieve the desired workability. Results indicated that at a 20% replacement rate, GCBA achieved 97.7% of the strength of pure cement, while coal fly ash reached 114.0%. Both materials exhibited similar volume stability in drying shrinkage tests. Still, GCBA demonstrated superior performance in reducing chloride ion permeability, with an RCPT test result of only 559 coulombs compared to 4108 coulombs for fly ash. This study suggested the feasibility of utilizing GCBA as a substitute for Portland cement in concrete, offering both carbon reduction and economic benefits.

Pozzolanic materials are used to partially replace ordinary Portland cement (OPC) in concrete or mortar. Pozzolanic properties depend on the physical and chemical properties of the pozzolanic materials [18]. Calcium silicate hydrate (C-S-H) and calcium hydroxide (Ca(OH)_2_) are the products of Portland cement hydration. Approximately 50% C-S-H and 15% to 25% calcium hydroxide are typically found in hydrated Portland cement paste. C-S-H, often called glue or gel, is the primary cementitious binder in hardened Portland cement concrete. Calcium hydroxide possesses fewer cementitious qualities and does not significantly increase the concrete’s strength. The pozzolanic material reacts with the calcium hydroxide to form additional C-S-H gels. This C-S-H composition may differ from Portland cement hydration; however, it increases the concrete’s strength and decreases its permeability [19].

About 730 million tons of CBA are generated in coal-fired power plants worldwide [14]. Only 41% percent of the CBA and 61% of the CBS produced in the United States were utilized in 2021 [20]. CBA is generally disposed of in landfills and ponds, contaminating the water source [16,17,21]. Using these materials as cementitious materials will reduce waste in landfills, preserve natural resources, reduce health hazards, and benefit the economy [14,16,17,21,22]. Fly ash is currently used as partial cement replacement in concrete because of its pozzolanic properties, which improve the durability and strength of concrete [17,23,24]. Very few studies have focused on using GCBA as a cement replacement; therefore, this study examined using GCBA or GCBS as a cement replacement, which is possible due to its pozzolanic properties that improve concrete durability and mechanical properties [11,24,25,26,27].

Nanotechnology has been demonstrated to be the ideal approach for dealing with various concrete material problems. Many aspects of concrete are studied using nanotechnology. Alkali–silica reaction (ASR) is caused by alkali and silica in cement and aggregates. When cement is substituted with pozzolanic materials, the chance of and extent of the alkali silicate reaction are reduced. The incorporation of nanoparticles may alter concrete properties. As a finer material, the nanomaterial fills the pore space of the C-S-H gel structure and functions as a nucleus to form a stronger link with the calcium silicate hydrate gel particles, densifying the matrix. As a result, concrete’s mechanical and durability properties are improved [28].

Selvasofia et al. [29] examined the use of nano-TiO_2_ and nanoclay in concrete. For the initial phase, the authors substituted cement with 1% to 4% nano-TiO_2_ to determine the optimal amount that provides the highest compressive strength. Compared to the control, 2% nano-TiO_2_ in concrete provided significantly higher compressive strength, flexural strength, and tensile strength at 7, 28, and 60 days. The fine aggregate was then replaced with 1% to 4% nanoclay while the nano-TiO_2_ content remained at 2%. The article revealed that concrete containing 2% nano-TiO_2_ replacing cement and 3% nanoclay replacing fine aggregates increased compressive, tensile, and flexural strength by more than 30% compared to the control.

Concrete that has cement replaced with coal byproducts has low early-age compressive strength due to the slower hydration of those particles; therefore, nanomaterials were added to increase the concrete’s early strength. Nanomaterials can enhance concrete’s mechanical and durability properties by allowing it to produce more C-S-H gel and improving the pore structure [29]. Adding GCBA and nanomaterials enhances the concrete’s early strength and improves concrete infrastructure sustainability [30,31]. This study investigates the GCBA and GCBS having unique chemical and physical properties from four different sources as a partial cement replacement in concrete. Mechanical properties like compressive strength, flexural strength, Modulus of Elasticity, Poisson’s ratio, and the RCPT test for durability properties have been tested and compared with cement concrete. Various studies on fly ash concrete with nanomaterial have been previously investigated [23,29,31]. However, the study of nanomaterials with GCBA and GCBS as partial cement replacements in concrete brings novelty to the research. The results of this research can potentially replace cement with local CBA in concrete, reducing waste in landfills, benefiting the economy, preserving natural resources, and reducing health hazards.

This study has several objectives. Firstly, the optimal amount of GCBA or GCBS that can replace cement, with and without nanomaterials, was determined by comparing their compressive strength to a cement-based control. Compared to the control, the effects of replacing cement with optimum amounts of GCBA or GCBS on the fresh, mechanical, and durability properties were evaluated. Finally, the impacts of the GCBA and GCBS properties on flow, water requirements, strength activity index (SAI), and compressive strength of a mortar were assessed.

## 2. Materials and Methods

### 2.1. Experimental Plan

**Figure 1:**
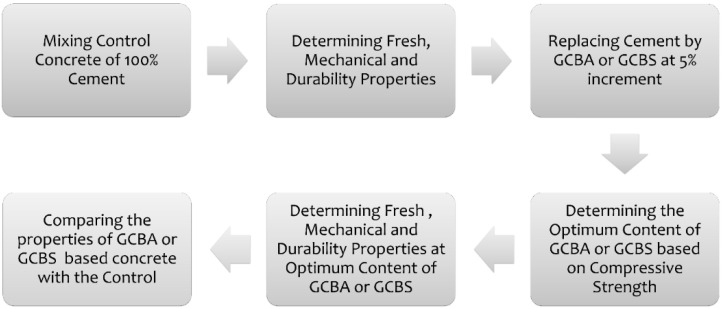
Experimental plan.

The experimental plan is illustrated in Figure 1. Cement-based concrete (control) was first mixed and tested for its fresh properties, mechanical properties, and durability. The compressive strength was then evaluated after replacing cement with GCBA or GCBS in 5% increments. The optimum GCBA replacement amount was concrete with a compressive strength comparable to or greater than the control. Then, the fresh, mechanical, and durability properties were evaluated. Nanoclay was also used to replace cement in increments of 0.5%, and the optimum content was determined. The GCBA and GCBS-based concrete properties with and without nanoclay were compared to the control. 

### 2.2. Material

#### 2.2.1. Fine and Coarse Aggregate, CBA, and CBS

Kost Materials and Strata Corporation provided Holcim Type I Portland Cement, fine aggregate, and coarse aggregate. The companies also provided information on coarse and fine aggregate physical properties, such as fineness modulus, specific gravities, and absorption. Following the American Society for Testing and Materials (ASTM) and American Association of State Highway and Transportation Officials (AASHTO) standards, the lab measured the same properties. The results were similar to those provided by the companies, as illustrated in Table 1. Specific gravity and absorption were measured in accordance with the AASHTO T 84 standard [32] for fine aggregates and the AASHTO T 85 standard [33] for coarse aggregates. The fineness modulus of the fine aggregate was calculated following the ASTM C136 standard [34].

**Figure 2:**
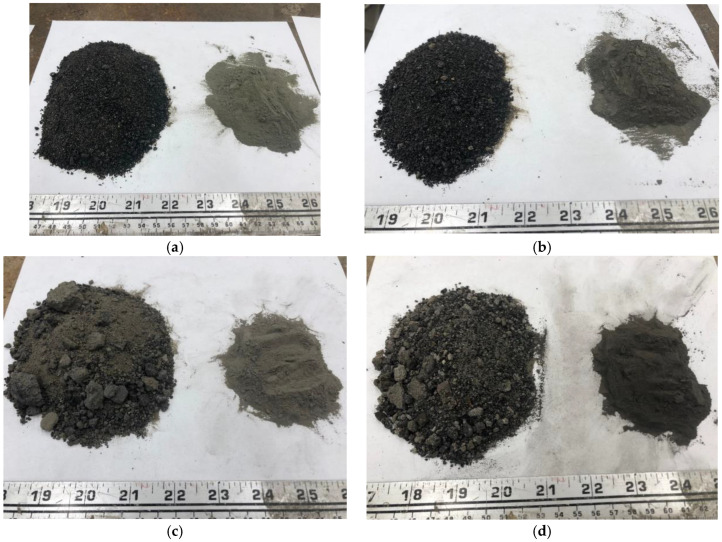
CBA, CBS, GCBA, and GCBS: (**a**) CBS and GCBS from MR Young Power Plant; (**b**) CBS and GCBS from Coyote Station; (**c**) CBA and GCBA from Coal Creek Station; (**d**) CBA and GCBA from Leland Olds Power Plant.

Four power plant stations supplied coal byproducts for the project. Great River Energy (Coal Creek Station) and Basin Electric Power Cooperative (Leland Olds Power Plant) supplied CBA, and Minnkota Power Cooperative (Milton R. Young Power Plant) and Otter Tail Power Company (Coyote Station) supplied CBS. The physical properties of the CBA and CBS were determined using the standards for fine aggregates. The physical properties of the CBA and CBS were similar to the fine aggregates (see Table 1). The absorption of the CBA was higher than that of the CBS due to its porous nature. The specific gravity of the CBS was higher than the CBA since it is denser and glassier. Figure 2 depicts the CBA and CBS with their respective GCBA and GCBS.

#### 2.2.2. GCBA and GCBS

The physical properties of the cement, GCBA, and GCBS, including Sieve No. 325 fineness, Blaine fineness, and Specific Gravity, were determined in the lab following the ASTM C40 [35], ASTM C204 [36], and ASTM C188 standards [37], respectively, as shown in Table 2. Two companies ground the CBA and CBS, denoted as 1 and 2. CC1 and CC2 were designations given to the GCBA received from the Coal Creek Station, which was ground by Company 1 and Company 2, respectively. L1 and L2 denote the GCBA received from the Leland Olds Station, ground by Company 1 and Company 2, respectively. Similarly, MR1 and MR2 denote the GCBS received from the Milton R. Young Station, and CO1 and CO2 denote the GCBS received from the Coyote Station. These acronyms are also used for the mortar based on each material.

Ecomaterial Technologies provided the chemical properties of the GCBA and GCBS. The cement supplier provided the chemical properties of the cement. The research team determined the loss of ignition (LOI) and moisture content of the GCBA and GCBS following the ASTM C311 standard [38]. The LOI value for the GCBA from the Leland Old Station was high, whereas it was negative for the GCBS from the Coyote and MR Young Stations. The negative LOI value for the GCBS could be caused by the oxidation of magnetite to hematite when heating at 750 °C, which increases the weight instead of decreasing it [39]. The oxidation of the sulfur present in the slag could be the reason for the negative LOI [40]. The chemical properties, LOI, and moisture content are listed in Table 2. The LOI for the GCBA from the Leland Old Station was 9.8%, which does not meet the chemical requirement criteria for fly ash: less than 6% as per the ASTM C618 standard [41]. GCBA and GCBS fineness is dependent on the grounding process. The Sieve No. 325 fineness for the GCBA and GCBS, except the L1 sample from the Leland Old Station ground by Company 1 and the CO1 sample from the Coyote Station ground by Company 1, failed to comply with the minimum requirements for the fly ash class, in accordance with the ASTM C618 standard.

According to the ASTM C618 standard, if the sum of oxides (SiO_2_, Al_2_O_3_, Fe_2_O_3_) is greater than 50% and calcium oxide is greater than 18%, it is Class C fly ash, and if calcium oxide is less than 18%, it is Class F fly ash. Therefore, based on the composition, GCBA from Leland Olds and GCBS from Coyote are categorized as Class C Fly Ash, whereas GCBA from Coal Creek and GCBS from MR Young are categorized as Class F Fly Ash.

#### 2.2.3. Nanoclay

Nanoclay was used as the nanomaterial to replace cement in this research. Nanoclay or montmorillonite clay has a layered structure with a thickness of about 1 nm and a lateral dimension of around 100 nm. In the cement matrix, nanoclay can function as a filler, reducing the holes and increasing the surface area that can undergo chemical reactions. In addition to increasing the concrete’s resistance to cracking, this can increase the material’s strength and stiffness. The energy X-ray study has shown that the main components of nanoclay are carbon, aluminum, silicon, and oxygen. Nanoclay can act as a reinforcing agent and filler in concrete [42].

### 2.3. Mix Design

#### 2.3.1. Mortar Mix Design

The mortar mix design was adopted from the ASTM C311 standard [38], as depicted in Table 3. GCBA and GCBS replaced 20% of the cement by weight. Graded standard sand was used as the fine aggregate. The water required for the GCBA-based mortar depends on the mortar’s flow. The mortar’s flow should be in the range of ± 5 of the control; 242 mL of water was used for the control following the ASTM C311 standard [38]. As mentioned earlier, the acronyms used for the various GCBA and GCBS materials have been used to design mortar mix types containing those materials. For example, CC1 will refer to the mortar containing 20% of CC1 GCBA.

#### 2.3.2. Mix Design of Concrete and Mixing

Mix design information was provided by the companies who provided the materials. Material from Strata Corporation and Kost Material were used in MIX1 and MIX2 mix designs. The research team also adjusted this design after the trial mix. The control mix contained coarse aggregates, fine aggregates, cement, water, and air-entraining admixture. The GCBA or GCBS content was increased at a rate of 5% to replace cement by weight to reach the optimum content. GCBA or GCBS was used to replace the weight of the cement to maintain the same water-to-cement ratio, keeping the water-to-cement ratio constant at 0.45 for the MIX1 and 0.42 for the MIX2. The mix design was corrected for aggregate moisture before mixing. The suppliers provided fine and coarse aggregate properties, which were also tested by the laboratory, with similar results (see Table 2). The properties from the suppliers were used to perform moisture corrections since the aggregate samples they used were more representative of the source. Table 4 lists the MIX1 and MIX2 mix designs used for one cubic meter of concrete. The concrete was mixed following the AASHTO R39 standard [43] using a mechanical mixer. Only one mixer was used during the project to reduce the variability caused by mixer rotation. Nanoclay replaced 2% and 2.5% of cement in the Coal Creek GCBA-based concrete for MIX1.

### 2.4. Testing

#### 2.4.1. Mortar Flow and Compressive Strength

The mortars’ compressive strength was tested following the ASTM C109 standard [44]. Mortar flow was measured using the ASTM C1437 standard [45] after mixing, according to the procedure given by ASTM C305 [46]. The mortar was then mixed for 15 s at medium speed, and mortar cubes of side 5 cm were created following the ASTM C109 standard. The cubes were kept in a moist room immediately after casting, removed from the mold after 24 h, and cured in saturated lime water. The mortar cubes were tested for compressive strength after 7 and 28 days of curing using a Universal Testing Machine (UTM). The strength capacity index and water requirements were only computed once the flow of the mix containing 20% GCBA was within ± 5 of the control, in accordance with ASTM C311 [38]. Water requirement is the percentage of water used in the 20% GCBA-based mortar mix compared to the control mix. A total of 6 cube samples for every mortar mix type were cast. Three of them were tested at 7-day curing, and the remaining three were tested at 28-day curing.

#### 2.4.2. Fresh Properties of Concrete

Fresh properties, such as slump, air content, and unit weight, were measured after the concrete was mixed. A Super Air Meter (SAM) was used to measure the air content using the pressure method following AASHTO TP118 [47]. Air content is the key factor for determining a concrete’s resistance to freezing and thawing. An increase in air content can lower compressive strength; therefore, it is necessary to maintain an appropriate air content for strength and durability. The slump was measured according to ASTM C143 [48] using the slump cone test. The unit weight of the fresh concrete was measured following ASTM C138 [49]. The control mix’s fresh properties were compared to the GCBA-based concrete to analyze the effect of replacing concrete with GCBA.

#### 2.4.3. Mechanical Properties of Concrete

Cylindrical specimens 10 cm in diameter by 20 cm in length were cast to test the compressive strength, splitting tensile strength, modulus of elasticity (MOE), and Poisson’s ratio following AASHTO R39 [43] using a UTM after 7, 28, 56, and 90 days of curing. All specimens were moist cured in the curing room following the AASHTO R39 standard [43]. Compressive strength was tested following AASHTO T22 M/T [50], the splitting tensile strength was tested following AASHTO T 198 [51], and MOE and Poisson’s ratios were determined according to the ASTM C469 standard [52]. Flexural strength was determined following the AASHTO T97 standard [53]. Beam specimens sized 15 cm × 15 cm × 53 cm were cast according to the AASHTO T23 standard [54]. At each testing period, three cylinders for the compressive strength test, two cylinders for the splitting tensile strength test, two cylinders for MOE and Poisson’s ratio test, and two beams for the Flexural Strength test were used.

#### 2.4.4. Durability Properties of Concrete by Rapid Chloride Permeability Test (RCPT)

**Figure 3:**
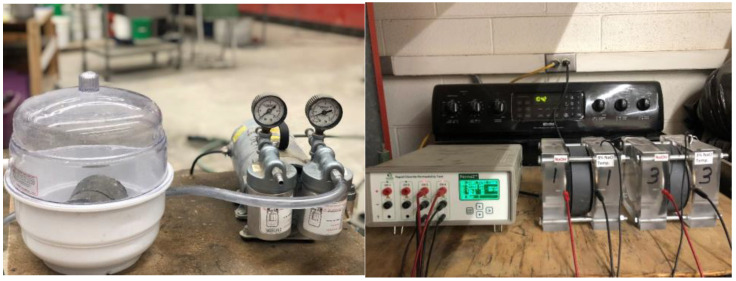
RCPT test of concrete.

The concrete’s permeability to chloride ions was measured using the Rapid Chloride Permeability Test (RCPT) following the ASTM C1202 standard [55], as shown in Figure 3. The RCPT test was conducted after 28 and 56 days of curing. The top 5 cm of the cylindrical specimen of 10 cm diameter and 20 cm length was sawed off and utilized for the RCPT test. For every RCPT test, two cylindrical specimens were sawed off at each testing period. The test assesses the concrete’s susceptibility to chloride ions, an important aspect when predicting the likelihood of steel reinforcement corrosion. The test’s findings are reported in coulombs, reflecting the total charge applied to the concrete during the test.

## 3. Results and Discussions

### 3.1. Mortar

#### 3.1.1. Flow, Water Requirement, and Strength Activity Index (SAI)

Table 5 lists the values for the Strength Activity Index (SAI), flow, and water requirements for the GCBA and GCBS samples. The 7- or 28-day SAI should be a minimum of 75% for fly ash use in concrete, as per ASTM C618. The GCBA or GCBS SAI is the percentage of compressive strength compared to the control. Only CC1, L1, L2, and CO1 met the SAI requirements. As specified in ASTM C618, the water requirements are a maximum of 105% for Class C and Class F fly ash, which was met by all GCBA and GCBS samples. The water needs for the L1 and L2 samples were greater than the other GCBA and GCBS samples (see Table 5), possibly because L1 and L2 had high LOI values of approximately 9.8%. A high LOI value implies a significant percentage of unburned carbon content, which absorbs water around the GCBA particles [13].

In our study, the water demand of GCBA or GCBS-based mortar was more dependent on the fineness and the LOI value. L1 and L2 were finer materials with higher LOI than the other GCBA and GCBS materials, indicating a higher surface area, which increased the water demand for the mortar pastes, reducing the flow.

A similar result was obtained from Abbas et al. [14], where the flow decreased as the incorporation of GCBA increased. The authors recommended using superplasticizers to improve workability. They concluded that grinding the CBA to a finer level will reduce the porosity and angularity of GCBA, resulting in increased flow properties.

Jaturapittakkul and Cheerarot [26] discovered that the water demand increased for the mortar containing sieved CBA, whereas it decreased for the mortar containing GCBA. The authors concluded that the increase in water demand for sieved CBA mortar was related to the high porosity of the CBA, which increased the water absorption, and the decrease in water demand for GCBA mortar is due to the smoother surface of GCBA compared to Portland cement.

The water requirements for the CC1 and CC2 samples were reduced to 99.2% compared to the control. The water requirements for the MR1, MR2, CO1, and CO2 samples were 100%, but the flow was higher than the control for the same amount of water. The fineness of all GCBA and GCBS samples was lower than the cement (see Table 2), indicating less surface area and water required for workability. The LOI values for these GCBA and GCBS samples were also less than 1.5%, indicating less unburned carbon.

#### 3.1.2. Mortar Compressive Strength

**Figure 4:**
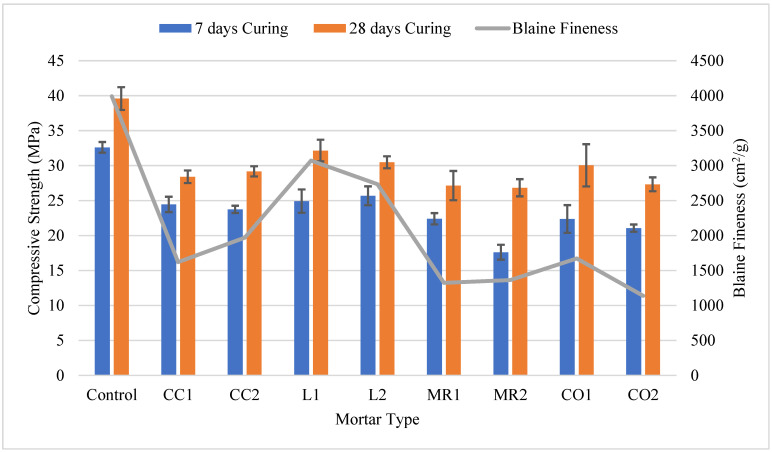
Comparison of compressive strength of mortar and Blaine fineness.

Figure 4 depicts the mortar’s compressive strength and the Blaine fineness for all GCBA and GCBS samples. The control’s compressive strength was always higher than the GCBA or GCBS-based mortar. The L1 sample had the highest compressive strength compared to all other GCBA and GCBS samples. The L1 sample was also the finest material among all other GCBA and GCBS samples. The specific gravity of the CBA sample from the Leland Station was the lowest, with a value of 2.11, which indicates it was a weaker material and easier to grind than other CBA and CBS samples. These results suggest that the compressive strength of the GCBA and GCBS-based mortars is directly related to the material’s fineness.

The MR2 sample had the lowest compressive strength compared to all other GCBA and GCBS samples. The MR2 sample’s fineness was higher than the CO2 samples; however, the MR2 sample’s chemical composition revealed a lower amount of CaO than CO2, which could be the reason for the mortar’s low early strength after 28 days. The MR1 sample had a higher compressive strength than the MR2 sample after 7 and 28 days of curing; however, the percentage increase in compressive strength was 17.5%, whereas it was 34.4% for MR2. The compressive strength of the MR1 and MR2 samples was nearly the same after 28 days. The Blaine fineness properties for MR2 were finer than MR1 (see Table 2). These results indicate that MR2 could possess better pozzolanic qualities than MR1 despite the lower compressive strength at 7 and 28 days.

Abbas et al. [14] discovered that the compressive strength of mortar with 20% GCBA had an SAI value of 86% at 28 days, slightly higher than the SAI value we received for L1, which is 81.2%. The Blaine fineness of the GCBA used in their research was 4355 cm^2^/g, which is finer than the 3072 cm^2^/g for L1 in our research. From this comparison, we can conclude that the fineness of GCBA plays an important role in compressive strength development.

Chuang et al. [17] discovered the same SAI value for 20% GCBA of 87% at 28 days. The SAI values exceeded the compressive strength of cement-based mortar at 180 days of curing with an SAI of 109%.

The CC2 sample had a lower compressive strength than the CC1 sample after 7 days of curing; however, the compressive strength of the CC2 sample was higher than the CC1 sample after 28 days of curing. The CC1 sample’s percentage increase in compressive strength from 7 to 28 days of curing was 13.9%, whereas the increase was 18.6% for CC2. The Blaine fineness for the CC2 sample was more than CC1. GCBA fineness is a critical property for boosting pozzolanic reactions. The pozzolanic reactions were minimal after 14 days of curing but rose after 28 days [25]. The chemical composition was the same for the CC1 and CC2 samples; however, the differences in material fineness resulted in a variation in compressive strength.

The L1 sample exhibited lower compressive strength than the L2 sample after 7 days of curing; however, the compressive strength was better after 28 days. The L1 sample’s percentage increase in compressive strength from 7 to 28 days of curing was 22.5%, whereas it was 15.7% for L2. The Blaine fineness and Sieve No. 325 fineness properties were finer for L1 than L2. These results are similar to those of the mortar based on the GCBAs from the Coal Creek Station. The compressive strength of the mortar increased after 7 days of curing, which is expected since the pozzolanic reactions are directly related to fineness [13].

The CO1 sample had a much higher compressive strength than the CO2 sample after 7 and 28 days of curing. The CO1 sample’s percentage increase in compressive strength from 7 to 28 days of curing was 25.5%, whereas it was 22.9% for CO2. The CO1 sample was significantly finer than the CO2 sample. The fineness of the CO1 sample boosted the pozzolanic reaction, which raised the mortar’s compressive strength.

### 3.2. Concrete

#### 3.2.1. Optimum GCBA and GCBS Content

**Figure 5:**
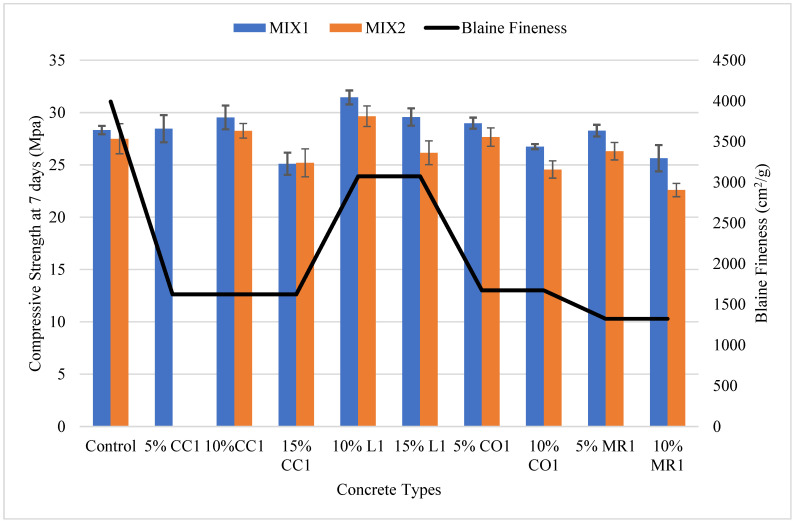
Compressive strength of the GCBA and GCBS-based concretes vs. control.

GCBA from the Coal Creek (CC1) and Leland Olds Stations (L1) and GCBS from MR Young (MR1) and Coyote Stations (CO1) were used to replace cement for the MIX1 partially. The Coal Creek GCBA-based concrete was evaluated by making a small control mix of 0.3 ft^3^ following MIX1 to make cylindrical specimens for 7-day compressive strength testing. The compressive strength of the GCBA-based concrete after 7 days of curing was the only value used to discover the optimum amount since it exhibits superior compressive strength at later ages due to an increase in pozzolanic reaction. Cement was then replaced with GCBA in 5% increments, after which 0.3 ft^3^ of concrete was made for every increment because the compressive strength could vary for different volumes of concrete mix. The specimens were tested for compressive strength, and the optimum GCBA content was determined. The optimum GCBA content was established as the mix with a compressive strength comparable to or greater than the control. The optimum content for the Coal Creek GCBA (CC1) sample was determined, then the other GCBA and GCBS-based concretes were mixed to determine the optimum content. The compressive strength of the CC1 concrete increased when 10% of the cement was replaced but decreased at 15%; therefore, the optimum content was established as 10%. The concrete also exhibited better compressive strength than the control at 10%. Figure 5 illustrates the 7-day compressive strength for all GCBA and GCBS-based concretes and the Blaine fineness for MIX1 and MIX2.

Abbas et al. [14] revealed that the optimum content for GCBA is 30% based on the strength of the mortar. Mangi et al. [3] discovered that the optimum content was 10% based on the compressive strength of concrete.

The optimum content for the MIX1 CC1 mix was 10%; therefore, for other GCBA and GCBS, cement replacement began at 10% and was changed to either 5% or 15% based on the 10% compressive strength result. The compressive strength of the L1 sample containing 10% cement replacement was the highest. The MR1 sample had the lowest compressive strength, at 10%. The L1 sample’s optimum content was established at 15% because the compressive strength was still higher than the control samples. The optimum replacement for the MR1 and CO1 samples was 5% since increasing the substitution to 10% did not improve the concrete’s compressive strength beyond the control. The Blaine fineness of the CO1 and CC1 samples was similar; however, the compressive strength for CC1 at 10% was higher than CO1. These results indicate that GCBA is more reactive than GCBS.

A 0.3 ft^3^ volume MIX2 control was mixed. The optimum content of the samples created using MIX1 and Coal Creek GCBA was approximately 10%; therefore, the research team began the MIX2 experiments by replacing 10% of the cement with GCBA or GCBS and then changed the amounts to 5% or 15%. The MR1 and CO1 GCBS materials replaced cement in 5% increments since their compressive strength at 10% replacement was lower than the control. The compressive strength when using L1 to replace 10% of the cement was the highest compared to all other GCBA and GCBS samples. The lowest compressive strength was recorded for the MR1 sample at 10%. The optimum content for L1 was established as 10%. Substituting 10% of the cement with MR1 and CO1 did not improve the concrete’s compressive strength beyond the control; therefore, the optimum quantity was determined as 5%.

#### 3.2.2. Fresh Properties of the Mixes Based on Optimum GCBA and GCBS

Specimens were prepared for all mechanical and durability tests once the optimum content was determined for the CC1 mix. This mix was similar in volume to the control mix. The fresh properties, such as slump, unit weight, and air content, were determined. Table 6 lists the fresh properties of the control mixes and those containing 10% CC1. The optimal MIX1 and MIX2 air content and slump values were lower than the control, and the unit weight was higher.

Mangi et al. [3] and Khan and Ganesh [22] found a similar workability result. Both papers observed that the workability of concrete decreased as the cement replacement by GCBA increased. The authors concluded that the workability of GCBA-based concrete depends on GCBA fineness. The finer the GCBA, the higher the reduction in workability due to increased surface area.

The air content of the GCBA-based concrete was lowered by almost 1.4% for both MIX1 and MIX2 mixes. However, Chuang et al. [17] noticed that the air content of GCBA-based concrete decreased as the GCBA content increased because an air-entraining admixture was not used in the mix. It has been discovered that incorporating fly ash or GCBA in concrete reduces the effectiveness of the air-entraining admixture [19]. So, it can be concluded that the dosage needs to be increased to increase the effectiveness of the air-entraining admixture in coal ash-based concrete.

The unit weight of GCBA-based concrete increased compared to the control. This contradicts the findings from Mangi et al. [3] and Chuang et al. [17], where the unit weight of concrete decreased with the incorporation of GCBA. This could be because we discovered decreased workability and air content in the GCBA-based concrete, which densified the concrete mix, and the unit weight was higher.

#### 3.2.3. Compressive Strength Comparison Based on Optimum GCBA and GCBS

**Figure 6:**
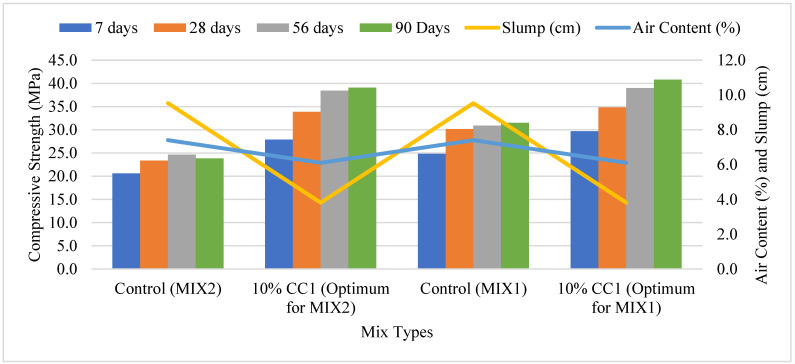
Compressive strength of the 10% CC1 vs. control mixes.

Figure 6 compares the compressive strength of the control to the compressive strength of the Coal Creek GCBA-based MIX1 and MIX2 concretes containing 10% GCBA. The two lines in the figure represent the slump and air content. The compressive strength of the concrete containing 10% CC1 was higher than the control after 7, 28, 56, and 90 days of curing. The workability and air content for both mixes with optimum CC1 content were lower than the control. Compressive strength decreases by 5–6% when air content is increased by 1% [19]; therefore, the higher compressive strength of the GCBA-based concrete can be attributed to the decrease in air content and workability.

Khan and Ganesh [22] discovered that the compressive strength of concrete with 10% GCBA replacement increased from the control by 14% at 56 days of curing. They concluded that the increase in compressive strength is due to more C-S-H (calcium silicate hydrate) gel formation by increased pozzolanic reaction with Ca(OH)_2_ at later ages.

Mangi et al. [3] found that increasing the amount of GCBA decreases the compressive strength of concrete. They compared the compressive strength of the control and GCBA concrete only after 28 days of curing and concluded that 10% GCBA replacement was the optimum content, which was close but still lower than the control.

#### 3.2.4. Comparison of Splitting Tensile Strength Based on Optimum GCBA and GCBS

**Figure 7:**
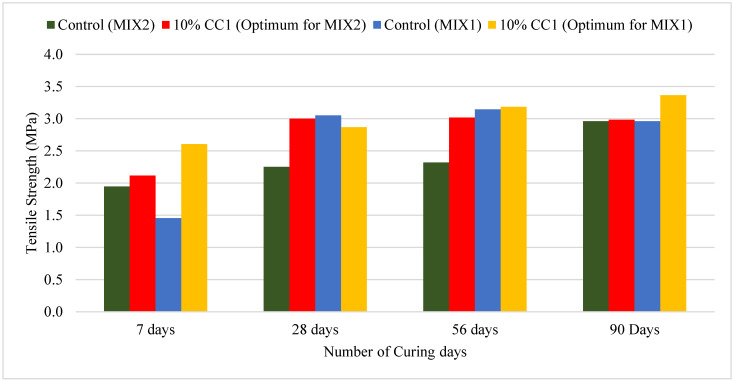
Tensile strength of the 10% CC1 vs. control mixes.

Figure 7 illustrates the splitting tensile strength of the control concrete compared to the tensile strength of the Coal Creek GCBA-based concretes with an optimum content of 10%. The splitting tensile strength of the MIX1 containing 10% CC1 was greater than the control at 7 and 90 days, similar at 56 days, and less at 28 days. The 10% CC1 mix’s tensile strength was only 8.2% of the compressive strength, and the control’s tensile strength was 10% after 56 days of curing. The compressive strength was much higher than the control; however, the tensile strength was approximately the same. Compressive strength is the concrete’s ability to resist compression; however, tensile strength is localized to a specific area, which could have resulted in lower or similar tensile strength compared to the control. Mangi et al. [3] discovered that the splitting tensile strength of concrete increased by 8% after replacing cement with 10% GCBA of Blaine fineness 3895 g/cm^2^. The splitting strength decreased when finer GCBA with Blaine fineness of 4638 g/cm^2^ was used.

Bheel et al. [4] performed splitting tensile strength using ground granulated boiler furnace slag (GGBFS) as cement replacement. They discovered that the splitting tensile strength was optimum at 10% replacement and the strength decreased at 20% replacement. They also revealed that the splitting tensile strength was higher than the control, with 30% CBA replacing fine aggregate. The mix with 10% GGBFS and 30%CBA gave the highest splitting strength compared to all the other mixes.

Ali et al. [6] discovered that the splitting tensile strength of concrete increased by 37% when 12.5% silica fume was used as a cement replacement and 30% CBA was used as a fine aggregate replacement.

The splitting tensile strength of the MIX2 containing 10% CC1 was greater than the control up to 90 days of curing. However, after 56 days of curing, the 10% CC1 mix’s tensile strength was only 7.8% of the compressive strength, whereas it was 9.4% for the control. The higher early tensile strength could also be related to the high amount of cement in the mix design, which initiated the pozzolanic reaction earlier than the MIX1.

#### 3.2.5. Comparison of Flexural Strength Based on Optimum GCBA and GCBS

**Figure 8:**
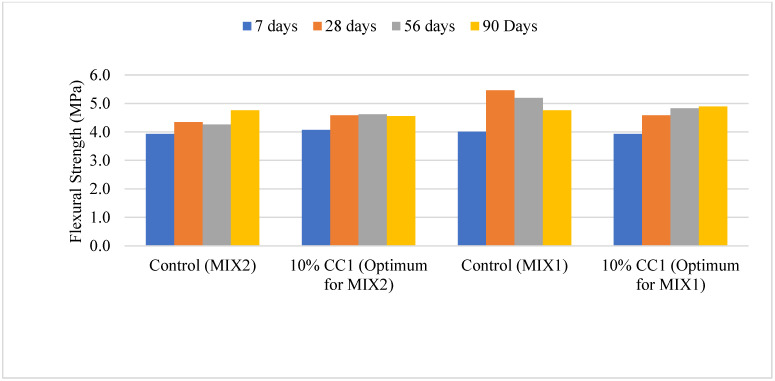
Flexural strength of the 10% CC1 vs. control mixes.

Figure 8 depicts the flexural strength of the MIX2 and MIX1 containing 10% CC1 mix compared to the controls. MIX1 containing 10% Coal Creek GCBA had a flexural strength lower than the control after 7, 28, and 56 days of curing; however, it was higher at 90 days. The control’s flexural strength was approximately 17% of the compressive strength, whereas it was only 12% for both mix designs containing GCBA. A concrete’s flexural strength is expected to be 10 to 20% of the compressive strength, and the measured values can vary depending on the testing method. Using GCBA in concrete might lower the bonding strength between the cement paste and aggregates. Flexural strength is carried from the cement paste to the aggregates; therefore, a lower bond strength might result in a lower flexural strength. These findings are comparable to the results from Mangi et al. [3], where the GCBA-based concrete’s flexural strength was lower than the control as the replacement percentage increased.

Abbas et al. [14] performed a flexural strength test of mortar with GCBA. The mortar’s flexural strength was high at 10% replacement, and it decreased at 30% to 40% replacement at all testing ages from 7 to 180 days of curing.

#### 3.2.6. MOE and Poisson’s Ratio Based on Optimum GCBA and GCBS

**Figure 9:**
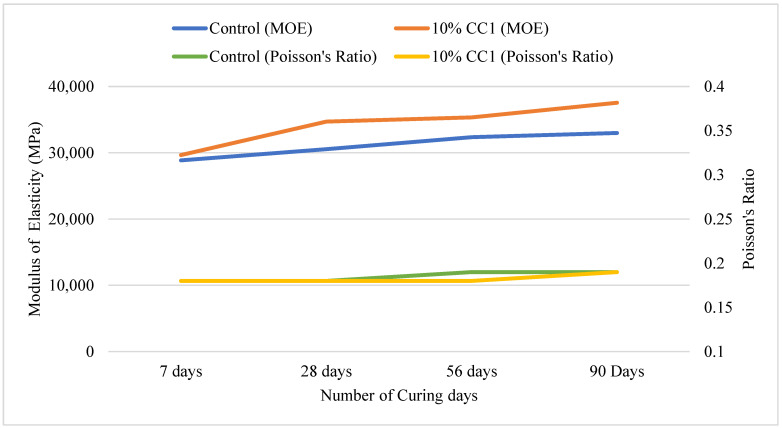
MOE and Poisson’s ratio for MIX1.

Figure 9 illustrates the MOE and Poisson’s ratio of MIX1 containing 10% CC1. The MOE of the GCBA-based concrete was higher than the control until 90 days of curing. It was approximately 8% higher than the control after 56 days of curing. The Poisson’s ratio was between 0.18 and 0.19 for both the control and the mix containing the optimum amount of GCBA, which was within the normal range of 0.15 to 0.25.

**Figure 10:**
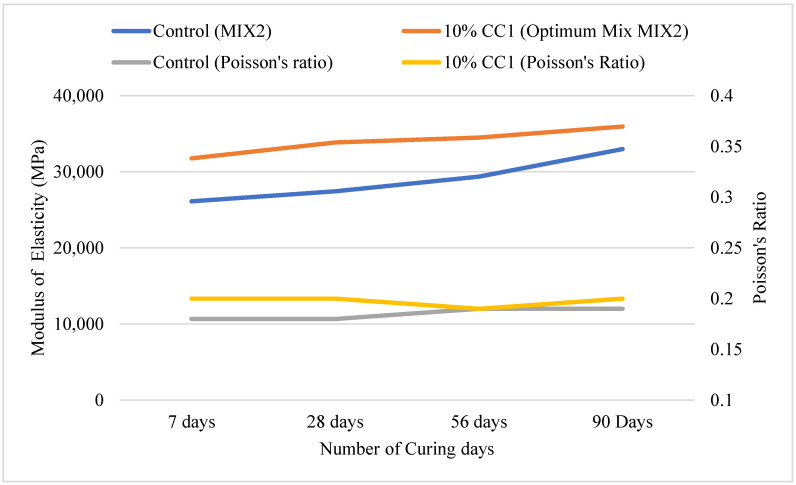
MOE and Poisson’s ratio for MIX2.

Figure 10 illustrates the MOE and Poisson’s ratio of the MIX2 containing 10% CC1. The MOE of the GCBA-based concrete was higher than the control until 90 days of curing. The MOE of the GCBA-based concrete was approximately 19% higher than the control after 56 days of curing. The Poisson’s ratio was similar. The Poisson’s ratio ranged from 0.18 to 0.20. The results indicated that the GCBA-based concrete’s MOE was higher than the control. A greater MOE suggests that it is stiffer and more rigid, which means it will deform less when loaded. A larger MOE can also increase load-carrying capacity, allowing concrete pavements to tolerate higher stresses and loads without failure.

#### 3.2.7. Comparison of RCPT Test Results for Durability Based on Optimum GCBA and GCBS

**Figure 11:**
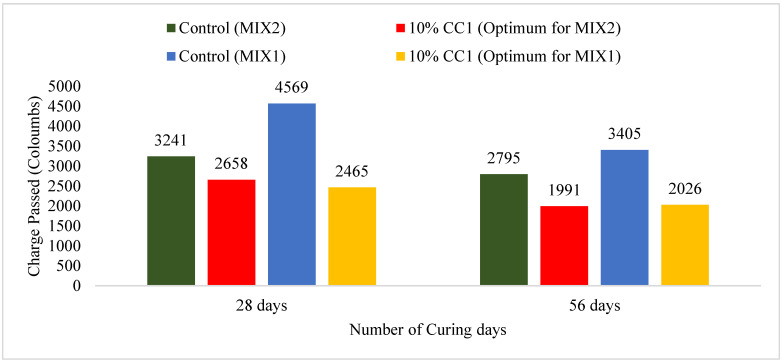
RCPT Test Results for the 10% CC1 vs. control mixes.

Figure 11 illustrates the charges passed in coulombs for the control mixes and the MIX1 and MIX2 containing 10% CC1. There is a significant difference in concrete permeability between the control and the MIX1 containing 10% CC1 after 28 days of curing. The charge passed in the control after 28 days was 84% higher than in the 10% CC1 MIX1. The 56-day permeability also indicates less charge passing through the GCBA-based concrete compared to the control. The charge passed in the control at 28 days was only 21% more than in the MIX2 containing 10% CC1. The control passed 40% more charge than the 10% CC1 MXI2 at 56 days. The MXI2 control’s permeability was lower than the MXI1 control because the amount of cement in the MXI2 was higher, which reduced concrete permeability. Kosmatka and Wilson [19] also revealed that using SCMs (Supplementary Cementitious Material) can reduce concrete permeability. GCBA-based concrete permeability decreases more at later ages due to more pozzolanic reactions taking place.

The result is comparable with the findings of Mangi et al. [27], where the authors performed a Rapid Chloride Permeability Test (RCPT) following the ASTM C1202 standard [55] at 28 days and 180 days for GCBA-based concrete and compared it with cement concrete. The authors discovered that including GCBA in concrete makes the concrete less permeable and reduces chloride penetration. They also concluded that the difference in permeability is greater at 180 days due to pozzolanic reaction at later ages.

The result also coincides with Chuang et al. [17] and Argiz et al. [12], which discovered that the chloride migration coefficient of concrete containing GCBA was lower than that of cement concrete and fly ash-based concrete. Argiz et al. [12] noticed that the increase in GCBA’s fineness decreases the penetration depth in concrete. Also, the chloride migration coefficient decreased as the replacement amount of GCBA increased from 10% to 25%. They concluded that GCBA can be used as a supplementary cementitious material (SCM) to increase chloride resistance.

Chuang et al. [17] noticed that the number of charges passing through the concrete consistently decreased with the increase in GCBA. However, for fly ash-based concrete, the number of charges passing decreased at 20% replacement but increased at 40% to 60% replacement. The authors concluded that GCBA-based concrete is more resistant to chloride ion penetration, which will increase the durability of concrete in chloride-susceptible areas.

#### 3.2.8. Optimum Content of Nanoclay with Coal Creek GCBA (CC1)

MIX1 mix design’s optimum CC1 GCBA content was 10% without nanoclay; however, this content can be increased by adding nanoclay. Selvasofia et al. [29] suggested that higher amounts of GCBA could be used if 2% of the cement was replaced with nanomaterials; therefore, 2% nanoclay was added, and the CC1 content was increased to 15%. The total cement replacement was 17%. The percentage of nanoclay and CC1 were then either increased or decreased based on the results, and the optimum nanoclay content was determined for the amount of GCBA added.

**Figure 12:**
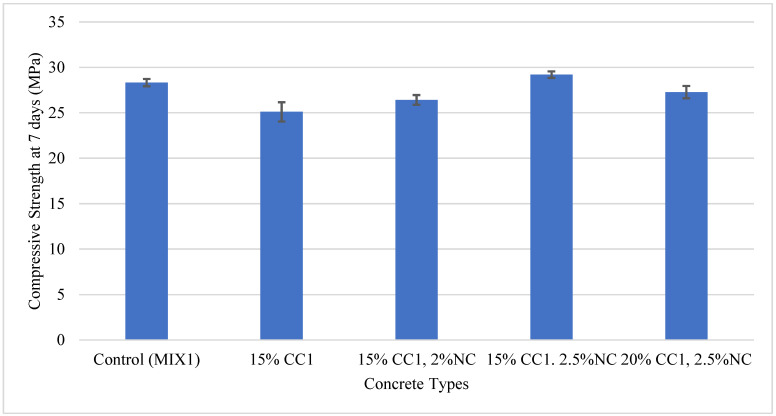
Compressive strength of the MIX1: GCBA with nanoclay vs. the control.

Figure 12 illustrates the 7-day compressive strength of MIX1 containing CC1 and nanoclay. The concrete containing 2% nanoclay (NC) and 15% GCBA had a lower compressive strength than the control; therefore, the next mix contained 15% CC1 and 2.5% NC, which resulted in higher compressive strength. MIX1 containing 20% GCBA and 2.5% nanoclay was created; however, the compressive strength was lower than the control. Nanoclay is expensive; therefore, experiments with 3% nanoclay and 20% CC1 were not performed. Due to this expense, the optimum content for nanoclay was established as 2.5%. Incorporating 2% nanoclay in the 15% CC1 concrete mix increased the compressive strength by approximately 5% compared to the 15% CC1 concrete. Adding 2.5% nanoclay increased the compressive strength of the 15% CC1 concrete by approximately 16%.

A similar result was obtained from Kumari et al. [23]. The authors investigated nanomaterials like nano-TiO_2_ and nano-CaCO_3_ to enhance the mechanical characteristics of concrete composed of fly ash. In the mix design, fly ash replaced 40% of the cement, followed by replacements of 0.5% to 3% of cement by nano-TiO_2_, nano-CaCO_3_, and combinations of both nanomaterials. Workability decreased with the increase in the amount of nanomaterials in the concrete. The concrete’s compressive strength and tensile strength significantly increased after adding nanomaterials. The highest compressive strength for nano-TiO_2_ was achieved at 3% replacement, whereas for nano-CaCO_3_ it was 0.5%. It was shown that the compressive strength increased when 1% nano-TiO_2_ and 1% nano-CaCO_3_ were used in place of 2% cement. The authors concluded that 2% replacement was the ideal content for both the nanomaterials and their combination after considering the durability properties on the RCPT test.

## 4. Conclusions and Recommendations

The following conclusions can be drawn from this study:

1. Mortar and concrete compressive strength depend on the fineness of the GCBA and GCBS. Finer GCBA and GCBS result in a higher compressive strength due to increased pozzolanic reactions;
2. GCBS has a lower LOI than GCBA. A high LOI indicates an increase in water demand in the mix;
3. Based on compressive strength, the optimum content of GCBA in concrete is 10% and 5% GCBS, which indicates that GCBA has more potential for replacing cement in concrete than GCBS;
4. Coal Creek Station GCBA-based concrete had better compressive strength and MOE at the optimum mix of 10%; however, there was no significant increase in tensile strength and flexural strength, which could be due to the weak bonding of the GCBA, and cement paste with the aggregates;
5. GCBA-based concrete was more resistant to chloride penetration;
6. Nanoclay increased the concrete’s early compressive strength. Adding 2.5% nanoclay increased the optimum content of the Coal Creek GCBA from 10% to 15%.

The following recommendations can be made based on the research:

1. GCBA or GCBS fineness should meet the specifications in ASTM C618 to obtain better results;
2. Using fiber in GCBA-based concrete must be studied because the flexural strength was lower than in cement concrete.

## 5. Future Works

There are future works related to the research as listed below:

1. Comparing the freeze and thaw durability of the GCBA-based concrete to the control;
2. Comparing the SAM number of the fresh concrete to the spacing factor of the hardened concrete to determine durability.

## Figures and Tables

**Table 1:** Physical properties of the fine and coarse aggregates, CBA, and CBS.

Physical Properties			Bulk Specific Gravity	Bulk SSD Specific Gravity	Absorption (%)	Fineness Modulus
Strata Corporation	Fine Aggregate	Lab	2.62	2.641	0.36	2.85
		Supplier	2.668	2.678	0.36	2.86
	Coarse Aggregate	Lab	2.605	2.634	0.91	-
		Supplier	2.660	2.690	0.91	-
Kost Materials	Fine Aggregate	Lab	2.651	2.65	0.38	2.74
		Supplier	2.668	2.678	0.36	2.86
	Coarse Aggregate	Lab	2.64	2.688	0.86	-
		Supplier	2.693	2.709	Not Received	-
CBA	Coal Creek	Lab	2.23	2.26	2.31	2.55
		Supplier	NA	NA	NA	NA
	Leland Olds	Lab	2.11	2.17	5.53	2.93
		Supplier	NA	NA	NA	NA
CBS	MR Young	Lab	2.23	2.26	2.31	2.55
		Supplier	NA	NA	NA	NA
	Coyote	Lab	2.11	2.17	5.53	2.93
		Supplier	NA	NA	NA	NA

**Table 2:** Physical and chemical properties of the GCBA, GCBS, and cement.

	Cement	GCBA (Coal Creek)		GCBA (Leland Olds)		GCBS (MR Young)		GCBS (Coyote)	
		CC1	CC2	L1	L2	MR1	MR2	CO1	CO2
**Physical Properties**									
Sieve No. 325 Fineness (% retained)	2.3	46.8	48.9	32.9	37.2	45.9	52.4	33.8	62.1
Blaine Fineness (cm^2^/g)	3992	1621	1970	3072	2735	1322	1364	1672	1138
Specific Gravity	3.104	2.665	2.674	2.556	2.632	2.717	2.753	2.904	2.899
**Chemical Properties**									
SiO_2_ (%)	19.8	51.87		36.61		47.9		35.96	
Al_2_O_3_ (%)	4.3	13.98		13.34		14.87		13.97	
Fe_2_O_3_ (%)	3.1	7.2		14.54		12.55		15.01	
Sum of Oxides (SiO_2_, Al_2_O_3_, Fe_2_O_3_)	27.2	73.06		64.5		75.32		64.95	
Cao (%)	64	15.05		20.06		12.34		18.8	
MgO (%)	2.5	4.63		6.26		4.48		5.35	
SO_3_ (%)	3.3	0.66		2.66		0.21		0.31	
LOI (%)	1.5	1.5		9.8		−1.4		−0.5	
Moisture Content (%)	0.5	0.3		1.5		0.1		0.1	
Class of Fly Ash (ASTM C618)	NA	F		C		F		C	

**Table 3:** Mortar Mix Design.

Mortar Mix Type	Cement (g)	Graded Standard Sand (g)	CC1 (g)	CC2 (g)	L1 (g)	L2 (g)	MR1 (g)	MR2 (g)	CO1 (g)	CO2 (g)	Water (mL)
**Control**	**500**	**1375**	-	-	-	-	-	-	-	-	**242**
CC1	400	**1375**	100	-	-	-	-	-	-	-	Water required for flow ±5 of the control mixture
CC2	400	**1375**	-	100	-	-	-	-	-	-	
L1	400	**1375**	-	-	100	-	-	-	-	-	
L2	400	**1375**	-	-	-	100	-	-	-	-	
MR1	400	**1375**	-	-	-	-	100	-	-	-	
MR2	400	**1375**	-	-	-	-	-	100	-	-	
CO1	400	**1375**	-	-	-	-	-	-	100	-	
CO2	400	**1375**	-	-	-	-	-	-	-	100	

**Table 4:** Concrete Mix Design.

Material (kg/m^3^)	Control		5% GCBA/GCBS		10% GCBA/GCBS		15% GCBA/GCBS		2% NC, 15% GCBA	2.5% NC, 15% GCBA	2.5% NC, 20% GCBA
Mix Design	MIX1	MIX2	MIX1	MIX2	MIX1	MIX2	MIX1	MIX2	MIX1	MIX1	MIX1
Cement	334.6	367.2	318	348.8	301.3	330.4	284.1	312.06	277.49	275.81	259.32
Coal Creek (GCBA)	-	-	16.61	18.39	33.22	36.78	50.43	55.17	50.43	50.43	66.92
Leland (GCBA)	-	-	16.61	18.39	33.22	36.78	50.43	55.17	-	-	-
Minnkota (GCBS)	-	-	16.61	18.39	33.22	36.78	50.43	55.17	-	-	-
Coyote (GCBS)	-	-	16.61	18.39	33.22	36.78	50.43	55.17	-	-	-
Nanoclay (NC)	-	-	-	-	-	-	-	-	6.69	8.37	8.37
Coarse Aggregate #1	972.9	1132.5	972.9	1132.5	972.9	1132.5	972.9	1132.5	972.97	972.97	972.97
Coarse Aggregate #2	74.1	-	74.16	-	74.16	-	74.16	-	74.16	74.16	74.16
Fine Aggregate	818.7	582.6	818.7	582.6	818.7	582.6	818.7	582.6	818.7	818.7	818.7
Water	150.6	154.2	150.6	154.2	150.6	154.2	150.6	154.2	150.7	150.7	150.7
Air Content (mL/m^3^)	115	239	115	239	115	239	115	239	115	115	115
W/C	0.45	0.42	0.45	0.42	0.45	0.42	0.45	0.42	0.45	0.45	0.45

**Table 5:** Flow, Water Requirements, and SAI of the GCBA and GCBS Mortar.

Sample	Water (mL)	Flow	Average Compressive Strength (MPa)		Strength Activity Index (%)		Water Requirement (%)
			7-Day	28-Day	7-Day	28-Day	
Control	242	93	32.61	39.59	100	100	100.0
CC1	240	91	24.45	28.40	75	71.8	99.2
CC2	240	91	23.75	29.18	72.8	73.7	99.2
L1	250	92	24.92	32.16	76.4	81.2	103.3
L2	250	91	25.69	30.48	78.8	77	103.3
MR1	242	94	22.40	27.14	68.7	68.6	100
MR2	242	98	17.61	26.83	54	67.8	100
CO1	242	98	22.38	30.05	68.6	75.9	100
CO2	242	96	21.06	27.33	64.6	69	100

**Table 6:** Fresh Properties of the 10% CC1 and Control Mixes.

	Slump (mm)	Air Content (%)	Unit Weight (kg/m^3^)
Control (MIX1)	95	7.4	2300
10% CC1 (Optimum for MIX1)	40	6.1	2332
Control (MIX2)	95	8.1	2281
10% CC1 (Optimum for MIX2)	40	6.7	2319

## Data Availability

The raw data supporting the conclusions of this article will be made available by the authors on request.
